# Enhancer of zeste homolog 2 (EZH2) in pediatric soft tissue sarcomas: first implications

**DOI:** 10.1186/1741-7015-9-63

**Published:** 2011-05-25

**Authors:** Roberta Ciarapica, Lucio Miele, Antonio Giordano, Franco Locatelli, Rossella Rota

**Affiliations:** 1 Department of Oncohematology, IRCCS, Ospedale Pediatrico Bambino Gesù, Roma, Italy; 2 Cancer Institute, University of Mississippi Medical Center, Jackson, MI, USA; 3 Sbarro Institute for Cancer Research and Molecular Medicine, Temple University, Philadelphia, PA, USA; 4 Department of Human Pathology and Oncology, Università of Siena, Siena, Italy; 5 Dipartimento di Scienze Pediatriche, Università di Pavia, Pavia, Italy

**Keywords:** EZH2, soft tissue sarcomas, epigenetics, methylation, methyltransferases

## Abstract

Soft tissue sarcomas of childhood are a group of heterogeneous tumors thought to be derived from mesenchymal stem cells. Surgical resection is effective only in about 50% of cases and resistance to conventional chemotherapy is often responsible for treatment failure. Therefore, investigations on novel therapeutic targets are of fundamental importance. Deregulation of epigenetic mechanisms underlying chromatin modifications during stem cell differentiation has been suggested to contribute to soft tissue sarcoma pathogenesis. One of the main elements in this scenario is enhancer of zeste homolog 2 (EZH2), a methyltransferase belonging to the Polycomb group proteins. EZH2 catalyzes histone H3 methylation on gene promoters, thus repressing genes that induce stem cell differentiation to maintain an embryonic stem cell signature. EZH2 deregulated expression/function in soft tissue sarcomas has been recently reported. In this review, an overview of the recently reported functions of EZH2 in soft tissue sarcomas is given and the hypothesis that its expression might be involved in soft tissue sarcomagenesis is discussed. Finally, the therapeutic potential of epigenetic therapies modulating EZH2-mediated gene repression is considered.

## Introduction

### Soft tissue sarcomas: a clinical challenge

Soft tissue sarcomas (STSs) are a group of heterogeneous malignant neoplasms thought to arise from molecular lesions occurring during the differentiation of mesenchymal stem cells (MSCs) [1]. STSs account for less than 1% of all adult tumors and for about 15% of all pediatric ones, with an estimated 10,520 new cases in the US in 2010 [2,3]. A series of chromosomal translocations have been identified as hallmarks of most STSs, such as t(X;18)(p11.2;q11.2) in synovial sarcoma, t(11;22)(q24;q12) in Ewing's sarcoma, t(2;13)(q35;q14) and t(1;13)(p36;q14) in alveolar rhabdomyosarcoma (RMS). These chromosomal rearrangements result in oncogenic fusion proteins that play direct roles in altering gene expression pattern in STS, promoting tumor aggressiveness. Because of their infiltrating behavior, only 50% of STSs are suitable for radical surgical resection. Moreover, a fraction of STSs are resistant to chemotherapeutic agents, especially the metastatic forms [4]. Doxorubicin, the drug used in standard single-agent chemotherapy protocols for the treatment of metastatic STS, results in only 20% to 25% response rates. Even the combination of doxorubicin with other agents, such as ifosfamide, has not dramatically improved the overall 5-year survival rate, which is no higher than 50% to 60% [4]. Nevertheless, chemotherapy represents the only viable strategy for palliation of symptoms in patients with metastatic disease, improving their quality of life [5]. New promising biological drugs, such as monoclonal antibodies to insulin-like growth factor receptor (IGFR), inhibitors of multityrosine kinases, and mammalian target of rapamycin (mTOR), have been introduced in STS clinical trials (Table 1) [4]. However, disease stabilization is still not seen in many patients, especially those affected by peculiar histological variants or showing poor-risk factors; it is reasonable to hypothesize that a combination of cytotoxic chemotherapy with targeted agents may be more appropriate to improve outcome in STS patients. A novel class of therapeutic targets is represented by epigenetic regulators, such as DNA methyltransferases (DNMTs), histone acetylases (HATs), histone deacetylases (HDACs), and histone methyltransferases (HMTs). Physiologically, all these enzymes work in concert for regulating gene expression by modifying the state of chromatin without altering DNA gene sequences in order to obtain a proper tissue determination. Increasing evidence demonstrates that they play key roles in human tumorigenesis, often being deregulated in terms of expression and/or activity and leading to silencing of essential regulators of cell proliferation and differentiation. Indeed, from comparative analyses, it appears that cancer genomes show different patterns of epigenetic modifications as compared to normal cells. Using inhibitory agents of all of these enzymes, it is possible to obtain pharmacological reversion of the tumor-specific gene expression profile, as well as reactivation of abnormally silenced tumor-suppressor genes in cancer cells [6]. Among these regulatory players, the histone methyltransferase enhancer of zeste homolog 2 (EZH2) is considered one of the most appealing epigenetic targets for therapy in human cancer [7].

**Table 1:** Targeted therapy clinical studies for soft tissue sarcoma (STS)

Biological molecular agents	Molecular target(s)	Clinical studies (phase) and clinical efficiency	Reference
Tyrosine kinase inhibitors (TKIs)			

Imatinib mesylate (IM)	c-Kit, PDGFR	Phase II study: 53.7% of patients with GISTs showed a partial response, 27.9% of patients showed stable disease, 13.6% of patients showed early resistance to imatinib, 5% of patients showed serious adverse events	[60]
		
		Phase III study: confirmation of the effectiveness of imatinib as primary systemic therapy for patients with incurable GIST. No advantages to higher dose treatment were reported.	[61]

Sunitinib malate (SM)	VEGF-R1, VEGF-R2, VEGF-R3, c-Kit, PDGFR, Flt-3, CSF1, neurotrophic factor receptors	Phase III study: 7% of patients with GIST showed partial response, 58% had stable disease, 19% had progressive disease; 27.3 weeks was the time-to-tumor progression for sunitinib vs 6.4 weeks for placebo. Progression-free survival was similar.	[62]
		
		Phase II study: 3-month progression-free rate of >40% for liposarcomas leiomyosarcomas	[63]
		
		Phase II study: 52% of patients showed metabolic stable disease, 20% of patients achieved stable disease for at least 16 weeks, 47% of patients achieved partial response	[64]
		
		Phase II study (current): SM activity in patients with certain subtypes of STS. The majority of these patients showed stable disease for 16 weeks.	[65]

Sorafenib	VEGF-R2, VEGF-R3, c-Kit, PDGFR, Raf/Mek/Erk	Phase II study: 14% of patients with angiosarcoma and 6% of patients with leiomyosarcoma had a response, 64% of patients developed intolerance at the drug dose used	[66]
		
		Phase II study: 78% patients with vascular tumors showed disease stabilization	[67]
		
		Phase II study (current): antitumor activity and acceptable toxicity profile in patients with antracycline-refractory STS	[68]

Pazopanib	VEGF-Rs	Phase II study: 12-week progression-free survival was reached by 44% patients with leiomyosarcoma, 49% of patients with synovial sarcomas, and 39% of patients with the other STS types	[69]

Nilotinib	BCR/ABL, c-Kit, PDGFR, CSF1R	Phase I study: nilotinib alone or in combination with imatinib was well tolerated and showed clinical activity in imatinib-resistant GIST patients	[70]

Mammalian target of rapamycin (mTOR) inhibitors			

Tensirolimus	mTOR	Phase II study: moderate toxicity and limited clinical activity	[71]

Everolimus	mTOR	Phase II study: acceptable toxicity. Limited clinical activity in heavily pretreated patients with bone and soft tissue sarcomas. The efficacy in imatinib-refractory and sunitinib-refractory GIST is promising.	[72]

Ridaforolimus (AP23573)	mTOR	Phase I study: safety of the drug; 27% of patients showed stable disease.	[73]
		
		Phase II study: 29% of clinical benefit rate. Prolongation of survival.	[74]
		
		Phase III study (current)	[75]

Insulin-like growth factor (IGF) receptor antibodies			

Figitumumab	IGF-1R	Phase I study: good tolerance of the drug	[76]

R1507	IGF-1R	Phase II study (current): R1507 is well tolerated. Significant activity has been observed in Ewing's sarcoma, RMS and OS with several dramatic responses seen in Ewing's sarcoma and RMS.	[77]

AMG479	IGF-1R	Phase I study: absence of severe toxicities	[78]

Mk-0646	IGF-1R	Phase I study (current)	[79]

CSF1 = colony stimulating factor 1; Flt = fms-related tyrosine kinase; GIST = gastrointestinal stromal tumor; OS = osteosarcoma; PDGFR = platelet-derived growth factor receptor; RMS = rhabdomyosarcoma; VEGF = vascular endothelial growth factor.

### The Polycomb group protein EZH2 in STS

**Figure 1:**
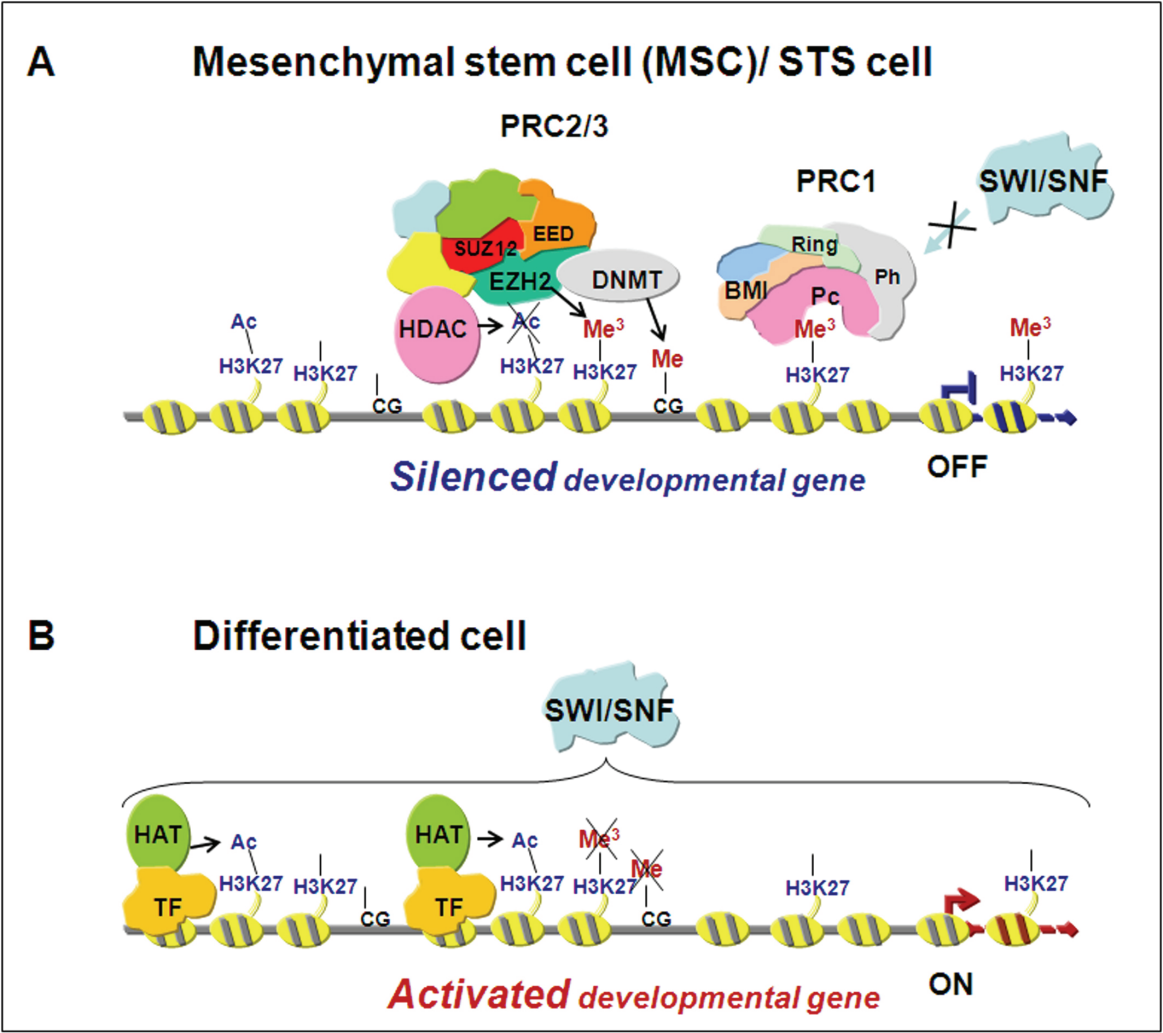
**Schematic representation of transcriptional gene repression by enhancer of zeste homolog 2 (EZH2)**. **(A) **In a mesenchymal stem cell or in a soft tissue sarcoma (STS) cell, EZH2 interacts with suppressor of zeste 12 (SUZ12) and embryonic ectoderm development (EED), the other core components of the Polycomb repressor complex 2 (PRC2) complex that is involved in the initiation of gene repression. By the methyltransferase activity of EZH2, histone H3 is methylated on K27 thus generating the epigenetic mark H3K27Me^3 ^that serves as a signal for the recruitment of PRC1 complex. PRC1 DNA binding prevents the access of antagonistic chromatin remodeling factors, such as the SWI/SNF complex, thus stabilizing the repressive state of the chromatin. The PRC2-associated activity of histone deacetylase (HDAC) and the interaction of EZH2 with DNA methyltransferases (DMNTs) allow a further compaction of chromatin by means of histone deacetylation and DNA methylation, respectively (synergism of epigenetic mechanisms). **(B) **During differentiation the level of EZH2 decreases with consequent reduction of PRC2 complex. H3K27 becomes hypomethylated and the SWI/SNF complex facilitates the DNA binding of tissue specific transcription factors (TF) that engage histone acetyltransferase (HAT) to allow initiation of transcription.

EZH2 is one of the Polycomb group (PcG) proteins, which repress expression of developmentally regulated genes that induce tissue differentiation, such as homeotic genes. PcG proteins help maintaining the undifferentiated, multipotent phenotype of the embryonic stem cell compartment [7-11]. In vertebrates, PcG proteins form two different groups of multiprotein Polycomb repressor complexes (PRCs), PRC1 and PRC2/3. EZH2 is the catalytic unit of the PRC2/3 complex, the part involved in the initiation of gene repression. EZH2 methylates lysine 27 of histone H3, thus generating the H3K27-trimethylated epigenetic mark that is recognized by the PRC1 complex for further, long-term chromatin modifications (Figure 1a) [8]. EZH2 is promptly downregulated during progenitor cell differentiation, becoming undetectable in adult specialized cells and tissues (Figure 1b) [12]. Conversely, EZH2 is abnormally overexpressed in a wide range of tumors as compared with corresponding normal tissues, its level of expression being correlated with cancer aggressiveness [7,13,14]. Moreover, the abundance of EZH2 molecules induces the formation of more repressor complexes and, by altering the balance between different PcG components, may lead to the formation of tumor-specific PRC complexes that show differential substrate specificities [15]. As a result, not only the general level of repression but also the specificity of repressed genes is changed. EZH2 has recently been found aberrantly expressed in aggressive and poorly differentiated breast and prostate carcinomas [13,14], as well as in STS [16,17]. EZH2 aberrant overexpression may be one of the molecular lesions occurring in differentiating mesenchymal stem cells (MSCs), which are thought to be the cells of origin of STS [1]. It has been proposed that the presence of EZH2 in tumors with embryonal features and stem-cell phenotype, such as STS, may explain their undifferentiated and immature character. In view of these data, EZH2 appears to be an attractive target for investigation in STS.

### EZH2 in RMS

**Figure 2:**
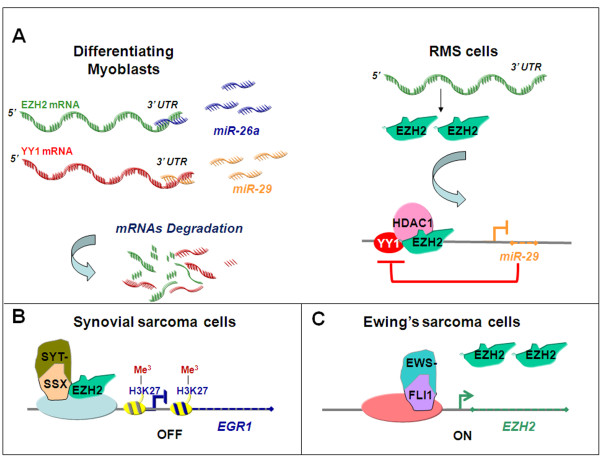
**Possible mechanisms of deregulation of enhancer of zeste homolog 2 (EZH2) in soft tissue sarcoma (STS)**. **(A) **In normal differentiating myoblasts (left panel) promyogenic miR-26a and miR-29 are normally expressed. MiR-26a and miR-29 target EZH2 and yin yang 1 (YY1) mRNAs, respectively, at the 3' untranslated region (UTR) to induce their degradation. Conversely, in rhabdomyosarcoma (RMS) cells (right panel) promyogenic miRNAs are downregulated and their loss of function is paralleled by the overexpression of EZH2 and YY1. YY1 recruits EZH2 to repress the expression of miR-29, establishing a negative regulatory feedback loop. **(B) **In synovial sarcoma, the chimerical transcription factor SYT-SSX engages EZH2 that leads to H3K27 trimethylation silencing tumor suppressor genes such as early growth response 1 (*EGR1*). **(C) **In Ewing's sarcoma the chimerical transcription factor Ewing sarcoma (EWS)/Friend leukemia integration 1 (FLI1) directly contributes to the maintenance of high level of expression of EZH2.

RMSs are a heterogeneous group of STSs characterized by features of skeletal muscle tissue and thought to be caused by abnormalities occurring during the course of myogenesis [18,19]. It prevalently affects pediatric patients and accounts for almost 50% of all STSs [20]. Classically, RMSs are histologically subdivided in two subtypes: the alveolar and embryonal forms. More recently, it has been reported that a diagnosis of alveolar RMS can be made only in the presence of two specific molecular aberrations, namely t(2;13)(q35;q14) and/or t(1;13)(p36;q14) chromosomal translocations resulting in PAX3-FKHR and the rarer PAX7-FKHR oncogenic fusion proteins, respectively [21]. These lesions have been found in about 20% of all RMSs and in about 70% of the RMSs with an alveolar histology [21,22]. True alveolar RMSs are often metastatic at diagnosis, show unresponsiveness to conventional therapy and have poor prognosis, the long-term survival rate being < 25% [23,24]. Fusion-negative RMSs include tumors with embryonal histology and the remaining part of RMSs with an alveolar histology [18]. Evidence for aberrant overexpression of EZH2 in RMS samples has been reported by Wang and colleagues [25] and by our studies in RMS cell lines and primary samples [16]. We have recently confirmed this finding in a large cohort of RMS specimens, documenting that overexpression of EZH2 is a hallmark of RMS, independently of the histological subtype [26]. It remains to be determined whether the level of EZH2 expression correlates with the presence of fusion proteins typical of the alveolar subtype. These results are consistent with the observation that in a physiological context EZH2 inhibits muscle differentiation of normal myoblasts by silencing muscle-specific genes [27]. Among these genes are those encoding for promyogenic microRNAs, such as miR-214 and miR-29. These belong to a class of small RNAs that inhibits the translation of selected mRNAs thus preventing their protein expression [25,28]. Mir-26a is another microRNA acting to post-transcriptionally repress EZH2 in normal myoblasts undergoing differentiation (Figure 2a left panel) [29]. During differentiation, miR-29 is induced and targets the PcG transcription factor yin yang 1 (YY1) mRNA promoting its degradation (Figure 2a left panel). In the absence of a myogenic stimulus and in RMS cells, EZH2 is recruited together with HDAC1 by YY1 to repress transcription of both myofibrillary genes [27,30] and miR-29 (Figure 2a right panel) [25]. Similarly, miR-214 is directly repressed by EZH2 in undifferentiated committed myoblasts and, in turn, it is able to bring about negative feedback on EZH2 during myogenesis by targeting its transcript [28]. A role for miR-26a and miR-29 in RMS pathogenesis was confirmed by recent studies [16,25]. We found that miR-26a is aberrantly downregulated in RMS cell lines and primary tumors as compared to non-tumor counterparts, and that miR-26a loss of expression is paralleled by an overexpression of EZH2 [16]. Similarly, miR-29 levels are reduced in tumor samples as compared with control muscle tissues. This finding can be interpreted considering that overexpressed EZH2 and YY1 are capable to repress miR-29 transcription in RMS cells (Figure 2a) [25]. In agreement with the above observations, it has been found that mi-R29 ectopic expression promotes RMS cell-cycle arrest, myogenic cell differentiation and tumor growth inhibition in a xenograft model [25]. Reduction of miR-29 levels had been previously reported in a small cohort of alveolar RMS [31]. Altogether, these findings provide evidence for a key role of EZH2-mediated epigenetic changes in RMS pathogenesis, which involve also mutual interactions with microRNAs.

### EZH2 in synovial sarcoma

Synovial sarcoma is a malignant cancer that affects prevalently young patients and represents almost 10% of all STSs [32]. It is characterized by the typical translocation t(X;18)(p11;q11) that generates the fusion between the synovial sarcoma translocation, chromosome 18 (*SS18 *or *SYT*) gene on chromosome 18 and either synovial sarcoma, X breakpoint 1, 2 or 4 (*SSX1*, *SSX2 *or *SSX4*) genes on the X chromosome [33]. Previously reported data showed that chimerical proteins SYT-SSX might disrupt gene expression mechanisms by functionally interacting with PcG proteins in synovial cells [34]. In particular, SYT-SSX2 fusion protein induces downstream target-gene deregulation through epigenetic mechanisms [35]. Recently, EZH2 has been found to mediate the effects of SYT-SSX activity. Specifically, SYT-SSX2 represses the expression of the tumor suppressor gene early growth response 1 (*EGR1*), a regulator of cell cycle, engaging EZH2 on the *EGR1 *promoter in synovial sarcoma cells (Figure 2b). *EGR1 *repression has been found to be associated with H3K27 trimethylation, and EZH2 and the PRC1 component BMI1 have been shown to directly bind its promoter, thus supporting the existence of a novel epigenetic mechanism of oncogenesis in synovial sarcoma [36]. This finding illustrates how a genetic lesion that generates an oncogenic trascriptional regulator might exploit EZH2 and other epigenetic regulators to sustain tumorigenesis.

### EZH2 in Ewing's sarcoma

Ewing's sarcoma is an embryonal malignancy characterized by the t(11;22)(q24;q12) translocation which generates chimerical Ewing sarcoma (EWS)/ETS fusion transcription factors. One of the most common fusion protein found in patients affected by this tumor is EWS/Friend leukemia integration 1 transcription factor (FLI1) [37]. EZH2 is expressed at high levels in Ewing's tumors [17]. Studying the influence of EZH2 downregulation on gene expression, Richter and colleagues found that EZH2 is responsible for the undifferentiated phenotype of Ewing's sarcoma by maintaining a *stemness *gene expression signature, inhibiting differentiation [17]. Strikingly, EWS/FLI1 has been found to induce the expression of *EZH2 *by direct binding to its promoter in both Ewing's sarcoma cell lines and human MSCs (Figure 2c) [17]. EWS/FLI1-dependent activation of *EZH2 *seems to be specific, because the other components of the PRC2/3 complex are not affected [38]. Notably, human MSCs seem to represent a permissive environment for the expression of EWS/FLI1, which induces features in these cells that recapitulate Ewing's sarcoma biology. This observation may implicate EZH2 as a coinitiator of Ewing's sarcoma [39]. Data from these studies offer an example of how a translocation-derived fusion product takes advantage of EZH2 recruiting this methyltransferase to drive tumor progression at the expenses of differentiation.

### Concluding remarks and future perspectives

Pediatric STSs, especially those metastatic at diagnosis, are highly aggressive tumors for which there is still an unmet medical need of more effective and less toxic therapeutic approaches. The role of the epigenetic regulator EZH2 in maintaining the embryonal cell phenotype of STS, its overexpression in these cancers and its functional interaction with many fusion proteins typical of STS, suggest that EZH2 may represent both a potential marker of undifferentiated precancerous cells and a reasonable candidate therapeutic target in STS. Increasing attention is focusing on epigenetic therapies that have provided promising results in clinical trials for some human tumors [40-42]. The clinical effectiveness of epigenetic therapies in human malignancies has been recently proved by the observation that, in a randomized phase III trial, the DNA hypomethylating agent azacytidine prolonged overall survival of myelodysplastic syndrome (MDS) patients compared to other standard therapies [43]. The potential efficacy of epigenetic therapy in STS is supported by preclinical studies employing HDAC inhibitors [36,44-46]. Many studies on cell culture and animal models indicate that diverse epigenetic processes synergize to control gene expression. Hence, different kinds of epigenetic drugs, such as DNA-demethylating agents and HDAC inhibitors, have been included in combination treatment protocols [40,47]. It is noteworthy that, in Ewing's sarcoma cells, HDAC inhibitor treatment *in vitro *induces downregulation of EZH2 [17], as more recently confirmed in glioma [48], gallbladder carcinoma [49] and acute myeloid leukemia [50]. Consistently, in preclinical models of different cancers, the antitumor effect of EZH2 inhibition, obtained through the methyltransferase inhibitor 3'-deazanoplanocin (DZNep), is enhanced by addition of HDAC inhibitors [51-53]. DZNep has been shown to act by causing depletion of PRC2 subunits with subsequent reactivation of PRC2-silenced genes [54,55]. In addition, it has been shown that the repressive function of EZH2 on gene expression is strengthened by the role of DNMTs, with which EZH2 physically interacts regulating their activity [56]. In this view, additional usage of DNMTs inhibitors in protocols targeting EZH2 might improve response in some tumor contexts. In turn, since HMTs are also active in non-proliferating cells, the inclusion of EZH2 inhibitors in combination regimens may overcome the ineffectiveness of DNMTs inhibitors in quiescent cells. On the other hand, it must be noted that, due to the complexity of molecular crosstalk involved in epigenetic control, the use of epigenetic drugs affecting a variety of molecular networks entails the risk of unforeseeable effects. For instance, despite their antiproliferative effects *in vitro*, treatments employing either HDACs or DNA methylation inhibitors have been recently reported to increase *in vivo *the invasive capabilities of RMS cells through upregulation of the prometastatic gene *Ezrin *[57]. Major questions remain open on the *in vivo *mechanism(s) of action of epigenetic drugs. Indeed, the clinical response to azacytidine in terms of prolongation of survival in MDS patients does not appear to be directly correlated with methylation of specific tumor suppressor genes, though methylation status has been shown to correlate with poor survival [58]. Even if future preclinical studies will better clarify the mechanisms of action of these drugs on gene expression, preclinical findings will need to be validated in humans [59].

Despite these unresolved questions, epigenetic therapy is a promising approach for targeted anticancer therapies in pediatric STS. Available evidence suggests that targeting the methyltransferase EZH2 may be potentially able to restore physiological patterns of gene expression in pediatric STS. In the future, modulation of EZH2 activity may provide a new line of intervention that could be combined with epigenetic drugs acting on other molecular targets and/or conventional cytotoxic agents to treat these aggressive pediatric tumors.

## Competing interests

The authors declare that they have no competing interests.

## Authors' contributions

RC and RR contributed equally to selection and discussion of the literature and the conception and preparation of the manuscript. FL, AG and LM contributed to the discussion on clinical implications and reviewed the manuscript. All authors read and approved the final manuscript.

## Pre-publication history

The pre-publication history for this paper can be accessed here:

http://www.biomedcentral.com/1741-7015/9/63/prepub
